# Bile acid, glucose, lipid profile, and liver enzyme changes in prediabetic patients 1 year after sleeve gastrectomy

**DOI:** 10.1186/s12893-020-00998-z

**Published:** 2020-12-14

**Authors:** Tsz Kin Mak, Shifang Huang, Bingsheng Guan, Hoyin Au, Tsz Hong Chong, Juzheng Peng, Fazhi Chen, Chuqiao Liang, Wanjing Lai, LongLam Ho, Cunchuan Wang, Jingge Yang

**Affiliations:** 1 Department of Gastrointestinal Surgery, First Affiliated Hospital of Jinan University, Guangzhou, 510630 China; 2 Department of Intensive Care Unit, First Affiliated Hospital of Jinan University, Guangzhou, 510630 China; 3 International School, Jinan University, Guangzhou, 510630 China

**Keywords:** Metabolic surgery, Prediabetes, Bile acid, Glucose change, Lipid change

## Abstract

**Background:**

Few articles have studied individuals with prediabetes after sleeve gastrectomy. Bile acid and lipid levels remain inconsistent in postbariatric patients. The purpose of this study was to explore bile acid, glucose, lipid, and liver enzyme changes in patients with different diabetes statuses who underwent sleeve gastrectomy. The impact of bariatric surgery and its potential benefits for prediabetic patients was also discussed.

**Methods:**

A total of 202 overweight and obese patients who underwent bariatric surgery in our hospital between January 2016 and October 2018 were retrospectively reviewed. Patients were divided into prediabetes (n = 32), nondiabetes (n = 144), and diabetes (n = 26) groups and analysed. Glucose and lipid data were collected from medical records at baseline and at each follow-up visit.

**Result:**

Significant improvements in body weight, glucose and lipid levels, and liver enzymes (P ≤ 0.05) in prediabetic patients were found throughout the first year postoperatively. Improvement in glycaemic control was first seen one month postoperatively, followed by persistent improvement in the next 12 months. Total bile acid (TBA) decreased, which was associated with ALT improvement in prediabetic patients 1-year post-surgery. There were no significant differences in HbA1c, glucose, or triglycerides (TGs) between prediabetic and T2DM patients or between prediabetic and nondiabetic patients at 12 months post-surgery.

**Conclusion:**

LSG is highly effective at interfering with glucose and lipid levels as well as total bile acid levels in prediabetic patients in the first year postoperatively. Thus, LSG is indeed an alternative for overweight and obese prediabetic patients.

## Background

Obesity is a worldwide health problem that affects children, adolescents, and adults and is accompanied by comorbidities such as hypertension, dyslipidaemia, type 2 diabetes, cancer, osteoarthritis, and sleep apnoea [1]. In particular, type 2 diabetes (T2DM) is a global pandemic, with a global prevalence of approximately 8.3% and is expected to rise to 10% by 2030 [2]. The correlation between T2DM and obesity has been well established. These two risk factors are steadily increasing in the general population, which may be the result of lifestyle changes [3]. While many focus on the risk of T2DM, few are concerned with prediabetes. Patients with a fasting plasma glucose (FPG) of 6.1 to 6.9 mmol/L and HbA1c of 6.0 to 6.4% are predicted to develop type 2 diabetes within 5 years [4]. Only a few studies have investigated patients with prediabetes after sleeve gastrectomy, which is a type of bariatric surgery.

Bile acid is a complex metabolic molecule that directly affects not only lipid metabolism but also intestinal peptide secretion and blood glucose control [5]. Bile acids are considered to be important mediators of weight loss and metabolic changes after bariatric surgery [6–9], and different proportions of bile acids are related to different characteristics of glucose metabolism [10, 11]. Most studies report increased bile acid concentrations after bariatric surgery [12, 13], but others reveal inconsistent changes in bile acid scores [14–16].

In our study, we explored the changes in glucose and lipid levels in overweight and obese prediabetic patients who underwent LSG, and the changes in bile acids observed in prediabetic patients are discussed.

## Methods

### Study design and patient selection

A retrospective observational study of a collected database was conducted in patients undergoing LSG in the Department of Metabolic and Obesity Surgery of the First Affiliated Hospital of Jinan University (Guangzhou, China) between January 2016 and October 2018. He et al. [17] found that comorbidity, mortality, and body composition data consistently supported the use of a lower BMI cutoff in Chinese patients than in white patients. Therefore, Chinese patients have their own standard BMI. The inclusion criteria were body mass index (BMI) ≥ 32.5 kg/m^2^ or ≥ 27.5 kg/m^2^ with one or more comorbid condition (hypertension, type 2 diabetes, dyslipidaemia, or OSA) that failed to be managed by lifestyle modification. The exclusion criteria were patients with a history of bariatric surgery or cholecystectomy. Moreover, patients with prediabetes were diagnosed according to CAD guidelines [18]. Patients with either impaired fasting glucose (IFG) or impaired glucose tolerance (IGT) and 6.0 to 6.4% HbA1c met the prediabetes diagnosis criteria. Patients were divided into three groups: T2DM, prediabetes and nondiabetes, according to their diabetes status. All participants were informed and consented to be involved in this study.

All patients completed a systematic routine examination before bariatric surgery and during each follow-up visit at 1, 3, 6, and 12 months postoperatively. The examination included routine physical examination, weight determination, obesity-related comorbidity investigation, and routine laboratory tests. Data, including age, sex, body weight, height, BMI, surgical method, HbA1c, insulin, C-peptide, fasting glucose, total bile acid (TBA), cholesterol (CHOL), triglyceride (TG), LDL-c, HDL-c, alanine transaminase (ALT), and aspartate transaminase (AST) were collected from medical records at baseline and during follow-up visits.

### Surgical technique

All surgical procedures were performed laparoscopically following standardized methodology by experienced surgical experts. The stomach was resected from the starting point for stapling, approximately 2–4 cm above the pylorus, followed by an entire fundus resection.

### Statistical analysis

The data are reported as the mean, standard deviation, and percentage. Statistical Product and Service Solution version 19.0 (SPSS 19.0, SPSS Inc., Chicago, IL) was used for data analysis. Student’s t-test or Mann–Whitney test was used to analyse continuous data. P ≤ 0.05 was considered statistically significant. The Pearson or Spearman coefficients were used for correlation analyses. Excel illustrates the line chart for listing variations of examined items.

## Results

A total of 220 patients undergoing sleeve gastrectomy presented in our centre from January 2016 to October 2018. Having excluded 8.9% (n = 18) of the individuals who presented with a history of choleocystomy, the remaining 202 individuals and their clinical characteristics are listed in Table 1. Sixteen percent (n = 32) of patients were diagnosed with prediabetes prior to bariatric surgery. Twelve out of 26 diabetics were newly diagnosed. Patients with a history of diabetes were managed with either oral anti-glycaemic agents or insulin alone or with a combination of oral agents and insulin. The medication was adjusted according to glycaemic status at each follow-up visit. The average BMI of the bariatric patients decreased significantly from 36.5 kg/m^2^ at baseline to 25.5 kg/m^2^ at 1 year post-surgery. Table 1 reveals that pre-op prediabetic patients had substantially greater BMIs than the other groups. Subgroup analysis of bile acid, glycaemic status, lipid profile and liver enzymes over time are shown in Table 2.

**Table 1:** The baseline of clinical characteristic of this study (excluded Cholecystomy)

	Pre-diabetes	Non-diabetes	Diabetes
Variables	Subjects (n=32)	Subjects (n=144)	Subjects (n=26)
Gender (male/female)	9 (28.1%)/23 (71.9%)	31 (21.5%)/113 (78.5%)	14 (53.8%)/12 (46.2%)
Age	29.9 ± 10.73	27.29 ± 8.61	30.92 ± 9.07
Weight (Kg)	107.8 ± 22.6	99.8 ± 19.8	111.3 ± 23.0
BMI	37.5 ± 5.2	36.2 ± 4.82	36.9 ± 4.01
HbA1c (%)	6.41 ± 1.09	5.39 ± 0.35	7.2 ± 1.14
Insulin (pmol/L)	26.5 ± 12.5	19.9 ± 12.3	23.8 ± 7.9
C-peptid e (ug/L)	3.8 ± 1.08	3.25 ± 1.31	4.39 ± 2.74
Glucose (mmol/L)	5.9 ± 1.03	5.08 ± 0.53	8.3 ± 2.67
TBA (umol/L)	4.3 ± 4.07	4.06 ± 4.69	3.77 ± 2.2
CHOL (mmol/L)	5.1 ± 0.99	4.9 ± 0.89	5.2 ± 1.47
TG (mmol/L)	2.3 ± 2.21	1.71 ± 0.91	2.23 ± 1.06
LDL (mmol/L)	3.2 ± 0.68	3.02 ± 0.74	3.5 ± 0.97
HDL (mmol/L)	0.99 ± 0.22	1.1 ± 0.29	1.0 ± 0.16
ALT (U/L)	69.2 ± 41.2	41.7 ± 40.89	74.4 ± 47.2
AST (U/L)	40.0 ± 22.7	26.8 ± 18.59	45.1 ± 26.8

**Table 2:** The variable at baseline and after bariatric surgery between the diabetes status of patient

	Pre-operative	1 Month	3 Months	6 Months	1 Year
*Weight (Kg)*					
Pre-diabetes	107.8 ± 22.6	95.3 ± 14.3^c,d,*^	87.8 ± 16.7^b.c,d,*^	73.6 ± 7.26^*^	74.2 ± 6.61^d,*^
Diabetes	111.3 ± 23.0	94.3 ± 15.96	85.16 ± 32.3	71.96 ± 9.59	78.2 ± 11.76
Non-diabetes	99.8 ± 19.8	84.9 ± 15.01	80.13 ± 15.3	73.85 ± 18.69	67.33 ± 10.84
*BMI*					
Pre-diabetes	37.5 ± 5.2	33.6 ± 4.3^c,*^	30.9 ± 5.02^*^	25.9 ± 2.48^*^	26.1 ± 2.53^c,d,*^
Diabetes	36.9 ± 4.01	33.5 ± 4.13	27.89 ± 3.99	24.46 ± 2.59	25.59 ± 3.33
Non-diabetes	36.2 ± 4.82	31.31 ± 4.15	29.21 ± 4.323	25.74 ± 3.76	25.34 ± 3.55
*HbA1c (%)*					
Pre-diabetes	6.2 ± 0.79^a,c^	5.7 ± 0.4^a,c,d,*^	5.3 ± 0.3^b,c,d,*^	5.2 ± 0.53^d,*^	5.3 ± 0.2^d,*^
Diabetes	7.2 ± 1.14	6.4 ± 0.56	5.3 ± 0.4	4.9 ± 0.4	5.08 ± 0.3
Non-diabetes	5.39 ± 0.35	5.2 ± 0.3	5.02 ± 0.3	5.09 ± 0.27	5.09 ± 0.23
*Insulin (pmol/L)*					
Pre-diabetes	26.5 ± 12.5^c^	13.9 ± 10.5^*^	10.7 ± 5.97^c,d,*^	7.3 ± 2.92^a,*^	5.7 ± 3.1^b,d,*^
Diabetes	23.8 ± 7.9	17.9 ± 10.99	12.05 ± 6.9	7.42 ± 5.03	6.75 ± 3.34
Non-diabetes	19.9 ± 12.3	9.3 ± 5.7	7.58 ± 4.07	11.39 ± 19.6	5.8 ± 3.11
*C-peptide (ug/L)*					
Pre-diabetes	3.8 ± 1.08^c^	2.7 ± 1.14^b,*^	2.5 ± 0.91^*^	2.4 ± 0.85^*^	1.7 ± 0.49^d,*^
Diabetes	4.39 ± 2.74	4.08 ± 1.68	2.75 ± 0.98	2.3 ± 0.99	1.97 ± 0.67
Non-diabetes	3.25 ± 1.31	2.44 ± 1.07	2.17 ± 0.82	2.4 ± 2.5	1.6 ± 0.6
*Glucose (mmol/l)*					
Pre-diabetes	5.9 ± 1.03^a,c^	5.12 ± 0.66^a,d,*^	5.1 ± 0.71^b,c,d,*^	5.1 ± 1.08^d,*^	4.9 ± 0.37^b,d,*^
Diabetes	8.3 ± 2.67	6.2 ± 1.26	5.52 ± 0.81	5.3 ± 0.36	4.7 ± 0.46
Non-diabetes	5.08 ± 0.53	4.96 ± 0.51	4.9 ± 0.47	4.9 ± 0.51	4.74 ± 0.39
*TBA (umol/L)*					
Pre-diabetes	4.3 ± 4.07	3.08 ± 2.37	2.3 ± 1.56	2.04 ± 0.87^a,b,*^	1.93 ± 0.65^*^
Diabetes	3.77 ± 2.2	2.6 ± 1.11	2.8 ± 1.57	4.33 ± 1.71	3.74 ± 3.0
Non-diabetes	4.06 ± 4.69	3.47 ± 3.43	2.57 ± 2.79	4.36 ± 7.28	2.64 ± 1.79
*CHOL (mmol/L)*					
Pre-diabetes	5.1 ± 0.99	4.48 ± 0.9	4.7 ± 0.67	5.2 ± 0.76	5.06 ± 0.72
Diabetes	5.2 ± 1.47	4.62 ± 0.39	4.83 ± 0.84	4.9 ± 0.7	4.71 ± 0.77
Non-diabetes	4.9 ± 0.89	4.37 ± 0.83	4.84 ± 0.87	4.9 ± 0.99	4.87 ± 0.81
*TG (mmol/L)*					
Pre-diabetes	2.3 ± 2.21	1.31 ± 0.41^*^	1.27 ± 0.35^*^	1.12 ± 0.25^c,*^	0.75 ± 0.19^*^
Diabetes	2.23 ± 1.06	1.8 ± 1.05	1.11 ± 0.29	1.17 ± 0.65	0.83 ± 0.19
Non-diabetes	1.71 ± 0.91	1.2 ± 0.42	1.11 ± 0.41	0.86 ± 0.23	0.86 ± 0.36
*LDL (mmol/L)*					
Pre-diabetes	3.2 ± 0.68	2.75 ± 0.78	3.06 ± 0.56	3.4 ± 0.58^c^	2.82 ± 0.43
Diabetes	3.5 ± 0.97	3.1 ± 0.34	3.04 ± 0.57	2.92 ± 0.76	2.7 ± 0.53
Non-diabetes	3.02 ± 0.74	2.73 ± 0.64	3.0 ± 0.69	2.93 ± 0.71	2.78 ± 0.51
*HDL (mmol/L)*					
Pre-diabetes	0.99 ± 0.22^c^	1.05 ± 0.8	1.0 ± 0.22	1.18 ± 0.21^*^	1.38 ± 0.17^*^
Diabetes	1.0 ± 0.16	0.87 ± 0.18	1.09 ± 0.28	1.16 ± 0.3	1.27 ± 0.27
Non-diabetes	1.1 ± 0.29	0.97 ± 0.18	1.09 ± 0.21	1.27 ± 0.24	1.39 ± 0.25
*ALT (U/L)*					
Pre-diabetes	69.2 ± 41.2^c^	48.6 ± 23.2^c^	26.2 ± 13.59^c,d,*^	15.3 ± 6.67^d,*^	12.86 ± 5.13^*^
Diabetes	74.4 ± 47.2	55.87 ± 32.64	31.21 ± 18.09	21.2 ± 11.76	18.9 ± 9.43
Non-diabetes	41.7 ± 40.89	34.1 ± 26.43	18.23 ± 11.56	18.77 ± 15.73	14.69 ± 5.31
*AST (U/L)*					
Pre-diabetes	40.0 ± 22.7^c^	38.6 ± 17.96^c^	21.9 ± 8.52^d,*^	18.02 ± 9.62^d,*^	17.9 ± 4.06^*^
Diabetes	45.1 ± 26.8	38.9 ± 19.8	25.28 ± 9.19	18.8 ± 8.55	18.1 ± 5.51
Non-diabetes	26.8 ± 18.59	26.9 ± 12.45	20.51 ± 16.24	18.63 ± 12.23	16.94 ± 3.68

*P≤ 0.05 versus pre-operative within pre-diabetes group

^a^P≤ 0.05 Pre-diabetes versus diabetes at the same time point

^b^P≤ 0.05 The change of concentration between pre-diabetes and diabetes

^c^P≤ 0.05 Pre-diabetes versus non-diabetes at the same time point

^d^P≤ 0.05 The change of concentration between pre-diabetes and non-diabetes

### Prediabetes

At 1 year post-surgery, patients with prediabetes exhibited a continuous significant reduction in weight, BMI, HbA1c, insulin, C-peptide, glucose, and TG (P ≤ 0.05), as revealed in Table 2. There was no significant difference in CHOL at 1 year post-surgery. The total bile acid concentration was significantly different at 1 year post-surgery. HDL was significantly increased 6 months post-surgery (P ≤ 0.05). AST and ALT were significantly decreased at 3, 6 and 12 months post-surgery. Improvement in glycaemic control and body weight was seen 1 month after surgery, with continuous improvement during the next 12 months. Additionally, Spearman’s rho, as shown in Table 3, revealed a correlation between the change in bile acid and ALT improvements.

**Table 3:** Correlation analyses in the pre-diabetes of fasting serum of total bile acids, fasting serum lipids and measures of glucose metabolism for 1 years

	Spearman’s Rhoa	P value
Change from baseline to 1 year	Change in total bile acid concentrations	
Gender	− 0.27	0.47
Age	− 0.38	0.32
Weight (Kg)	− 0.12	0.78
BMI	− 0.43	0.29
HbA1c (%)	0.16	0.71
Insulin (pmol/L)	− 0.02	0.95
C-peptide (ug/L)	− 0.21	0.61
Glucose (mmol/L)	0.09	0.81
CHOL (mmol/L)	− 0.15	0.7
TG (mmol/L)	0.50	0.16
LDL (mmol/L)	− 0.58	0.27
HDL (mmol/L)	− 0.40	0.31
ALT (U/L)	0.67	0.04*
AST (U/L)	0.38	0.31

* P ≤ 0.05 Statistically significant correlation between the change in bile acid and ALT

### Comparison between patients with different glucose statuses

**Fig. 1:**
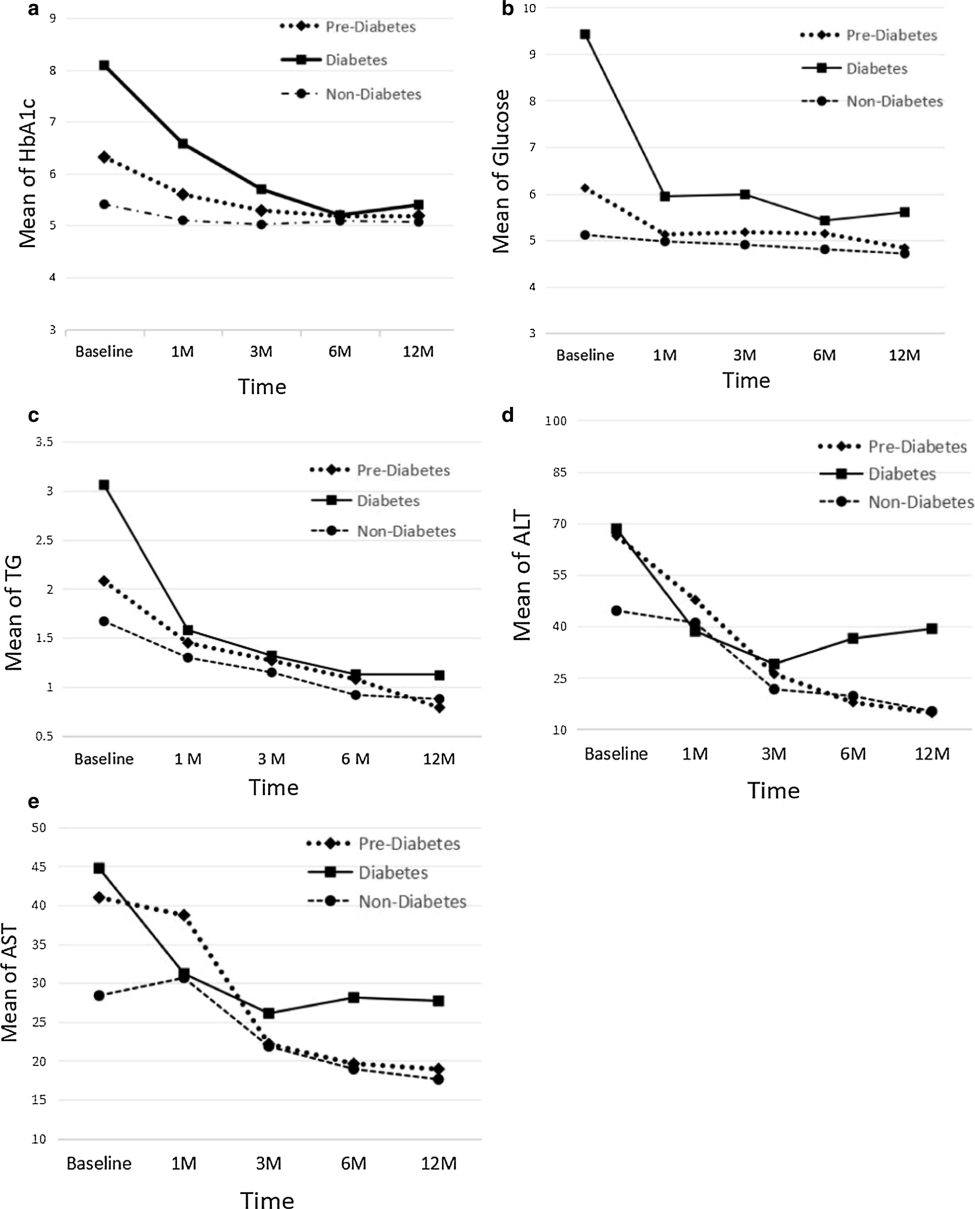
The concentration of HbA1c (**a**), glucose (**b**), TG (**c**), ALT (**d**) and AST (**e**) in different diabetes status of patients before, 1 month, 3 months, 6 months and 12 months after surgery

Prior to bariatric surgery, there was a significant difference in HbA1c and glucose between prediabetes and diabetes patients at the significance level when P ≤ 0.05, as described in Table 2. Six months after surgery, glucose control in both groups was notably improved (Fig. 1a, b). There were no significant differences in HbA1c and glucose between any of the groups at 12 months postoperatively. The change in glucose between prediabetes and type 2 diabetes subjects was significantly different 1 year postoperatively (P ≤ 0.05). Lipid changes exhibited no significant differences between prediabetes and diabetes patients after bariatric surgery (Table 2 and Fig. 1c). The liver enzymes were improved postoperatively, but the value of prediabetes patients was higher than that of nondiabetic patients at baseline (Fig. 1).

The preoperative BA, CHOL, LDL and TG levels of prediabetic and nondiabetic patients were not significantly different, as revealed in Table 2. At 6 months after surgery, glucose control, the lipid profile and liver enzyme levels were remarkably improved in both groups (Fig. 1). Body weight, glucose and lipid changes in the prediabetes patients shared some degree of similarity with the nondiabetic group at 1 year postoperatively (Table 2 and Fig. 1). The changes in HbA1c and glucose between prediabetes and nondiabetic subjects were significantly different (P ≤ 0.05) at each interval following surgery. At 1 year post-surgery, prediabetes and diabetes patients exhibited glucose control at the normal standard level, and other examined items were also improved.

## Discussion

The majority of studies have indicated that bariatric surgery is an alternative for improving glucose and lipid metabolism. The remission of diabetes correlates with weight reduction [19]. Bariatric surgery is an excellent solution to diabetes remission and weight reduction in most cases [20]. However, only a few studies have researched the impact of sleeve gastrectomy on prediabetes. In this study, the changes in glucose, lipid profile, and bile acids in prediabetes patients who underwent LSG were explored and discussed.

A large meta-analysis [21] and recent observational studies [20] proved that improvement in glucose metabolism had a correlation with weight reduction. Similarly, a reduction in food intake capacity via bariatric surgery aided in weight loss herein, seen as continuous weight loss in nondiabetic patients, diabetic patients, and prediabetic patients during the course of a year postoperatively. In terms of glycaemic status, as expected, the initial HbA1c and fasting glucose of prediabetic patients appeared to be significantly different from those of nondiabetic patients alone and T2DM patients alone. Improvement in glycaemic control and body weight in prediabetes and diabetic patients began at the first month postoperatively and persisted in the next 12 months. This result corresponded with the findings of Rubio-Almanza et al., who suggested that bariatric surgery improves glycaemic control and obesity comorbidities in prediabetes patients [22]. In addition, nearly 70% of prediabetes cases in our centre were treated by LSG. Within the course of the study, remarkable improvement in glycaemic state was observed in both diabetic patients and prediabetic patients. LSG is therefore an effective alternative for prediabetes patients, who fail to be managed by lifestyle modification, to assist with both weight loss and glycaemic control, thus delaying and preventing the progression of T2DM [23]. The advantage of LSG is its minimal invasiveness, relatively low risk, few complications, and cost effectiveness; moreover, LSG is highly effective in delaying and preventing obesity and T2DM comorbidities and irreversible neurovascular complications in advance [24]. Hence, LSG shall be encouraged in pre-diabetics. However, our study was a short-term follow-up study, and long-term follow-up will be continued and necessary, especially because these favourable results are related to weight loss.

Epidemiological and clinical studies have not only shown that HDL-C is negatively correlated with the incidence of atherosclerosis-related disease but also have recently suggested that some effects of bariatric surgery take place rapidly after surgery, as they may involve increased HDL levels [25–27]. Similarly, HDL, ‘good cholesterol’, was significantly increased in prediabetic patients after 6 months of the LSG operation. Increased HDL is thought to be associated with improved hepatic insulin sensitivity [28]. This may explain the improved glycaemic status after bariatric surgery. Further research may be necessary to reveal the mechanism by which HDL affects glucose metabolism.

Bile acids are derivatives of cholesterol synthesized in hepatocytes; thus, bile acid synthesis is a regulator of body cholesterol. The initial mean concentration of TBA in diabetic and prediabetic patients was 3 µmol/L higher than that in Western patients [29–32]. The cause of high bile acid levels remains unclear. Fatty liver, race, glycaemic control or lipid profile may possibly contribute to the cause, resulting in compensatory bile acid secretion. Bile acid levels in prediabetic patients were significantly reduced by the first year postoperatively. Some studies have indicated that an increase in bile acids strongly correlates with an improvement in glucose and lipid metabolism [29, 30], but Robert et al. [32] did not support the hypothesis that bile acids are key mediators of the early increase in postprandial GLP-1 and PYY secretion for improvement in glucose metabolism in postbariatric patients. In addition, Hilde Risstad et al. [31] indicated from data that bile acids had no significant change in 1-year postbariatric patients but significantly increased after 5 years. It has been proven that bile acids are not key mediators for the improvement of glucose in early postbariatric patients. Similarly, our results do not suggest that bile acids contributed to the rapid improvement in glycaemic control seen shortly after surgery.

Regarding lipid changes, this study revealed that triglycerides (TGs) continued to significantly decrease. Because bile acids are synthesized and secreted by hepatocytes, liver function directly affects the synthesis of bile acids. Clinically, ALT and AST are sensitive indicators of liver damage. ALT and AST were reduced in this study, indicating an improvement in liver function after metabolic surgery. Additionally, there is a correlation between the change in bile acid and ALT in prediabetes patients.

Some limitations should be noted in this study. First, bariatric surgery is still in the developing stages in China, and regular postoperative follow-up has not received close attention in some patients, resulting in a high rate of loss to follow-up. Thirty-five percent of the patients were followed up at 1 year postoperatively in this study; therefore, a small postoperative sample size was shown. In addition, our study was a short-term follow-up study, but long-term follow-up will be continued. Third, our results only reveal some clinical phenomena, and further research is necessary to consider prediabetes as a criterion for metabolic surgery and long-term effects after metabolic surgery.

## Conclusion

LSG is highly effective at improving glucose and lipid levels in prediabetes and diabetic patients. LSG is indeed an alternative for prediabetes and should be encouraged to prevent and delay the onset of diabetes and its irreversible neurovascular complications. Evidence from this study supports the phenomenon of total bile acid reduction during the course of the first year post-LSG surgery.

## Data Availability

The datasets generated and analysed during the current study are not publicly available as the data also forms part of an ongoing study but are available from the corresponding author on reasonable request.

## Abbreviations

SG: Sleeve gastrectomy
BMI: Body mass index
TBA: Total bile acid
TG: Triglycerides
ALT: Alanine transaminase
AST: Aspartate transaminase
CHOL: Cholesterol (CHOL

## References

[1] Guh DP, Zhang W, Bansback N, Amarsi Z, Birmingham CL, Anis AH (2009). The incidence of co-morbidities related to obesity and overweight: a systematic review and meta-analysis. BMC Public Health.

[2] Whiting DR, Guariguata L, Weil C, Shaw J (2011). IDF diabetes atlas: global estimates of the prevalence of diabetes for 2011 and 2030. Diabetes Res Clin Pract.

[3] Bastien M, Poirier P, Lemieux I, Despres JP (2014). Overview of epidemiology and contribution of obesity to cardiovascular disease. Prog Cardiovasc Dis.

[4] Heianza Y, Arase Y, Fujihara K, Tsuji H, Saito K, Hsieh SD, Kodama S, Shimano H, Yamada N, Hara S (2012). Screening for pre-diabetes to predict future diabetes using various cut-off points for HbA(1c) and impaired fasting glucose: the Toranomon Hospital Health Management Center Study 4 (TOPICS 4). Diabet Med.

[5] Thomas C, Pellicciari R, Pruzanski M, Auwerx J, Schoonjans K (2008). Targeting bile-acid signalling for metabolic diseases. Nat Rev Drug Discov.

[6] Kohli R, Bradley D, Setchell KD, Eagon JC, Abumrad N, Klein S (2013). Weight loss induced by Roux-en-Y gastric bypass but not laparoscopic adjustable gastric banding increases circulating bile acids. J Clin Endocrinol Metab.

[7] Gerhard GS, Styer AM, Wood GC, Roesch SL, Petrick AT, Gabrielsen J, Strodel WE, Still CD, Argyropoulos G (2013). A role for fibroblast growth factor 19 and bile acids in diabetes remission after Roux-en-Y gastric bypass. Diabetes Care.

[8] Ryan KK, Tremaroli V, Clemmensen C, Kovatcheva-Datchary P, Myronovych A, Karns R, Wilson-Perez HE, Sandoval DA, Kohli R, Backhed F (2014). FXR is a molecular target for the effects of vertical sleeve gastrectomy. Nature.

[9] Madsbad S, Dirksen C, Holst JJ (2014). Mechanisms of changes in glucose metabolism and bodyweight after bariatric surgery. Lancet Diabetes Endocrinol.

[10] Haeusler RA, Astiarraga B, Camastra S, Accili D, Ferrannini E (2013). Human insulin resistance is associated with increased plasma levels of 12alpha-hydroxylated bile acids. Diabetes.

[11] Wewalka M, Patti ME, Barbato C, Houten SM, Goldfine AB (2014). Fasting serum taurine-conjugated bile acids are elevated in type 2 diabetes and do not change with intensification of insulin. J Clin Endocrinol Metab.

[12] Cole AJ, Teigen LM, Jahansouz C, Earthman CP, Sibley SD (2015). The influence of bariatric surgery on serum bile acids in humans and potential metabolic and hormonal implications: a systematic review. Curr Obes Rep.

[13] Fouladi F, Mitchell JE, Wonderlich JA, Steffen KJ (2016). The Contributing role of bile acids to metabolic improvements after obesity and metabolic surgery. Obes Surg.

[14] Kohli R, Seeley RJ (2013). Diabetes: the search for mechanisms underlying bariatric surgery. Nat Rev Endocrinol.

[15] Dutia R, Embrey M, O'Brien CS, Haeusler RA, Agenor KK, Homel P, McGinty J, Vincent RP, Alaghband-Zadeh J, Staels B (2015). Temporal changes in bile acid levels and 12alpha-hydroxylation after Roux-en-Y gastric bypass surgery in type 2 diabetes. Int J Obes (Lond).

[16] Simonen M, Dali-Youcef N, Kaminska D, Venesmaa S, Kakela P, Paakkonen M, Hallikainen M, Kolehmainen M, Uusitupa M, Moilanen L (2012). Conjugated bile acids associate with altered rates of glucose and lipid oxidation after Roux-en-Y gastric bypass. Obes Surg.

[17] He W, Li Q, Yang M, Jiao J, Ma X, Zhou Y, Song A, Heymsfield SB, Zhang S, Zhu S (2015). Lower BMI cutoffs to define overweight and obesity in China. Obesity (Silver Spring).

[18] Canadian Diabetes Association Clinical Practice Guidelines Expert C, Goldenberg R, Punthakee Z. Definition, classification and diagnosis of diabetes, prediabetes and metabolic syndrome. Can J Diabetes 2013, 37 (Suppl 1):S8–11. 10.1016/j.jcjd.2013.01.011 24070969

[19] Taylor R (2013). Banting Memorial lecture 2012: reversing the twin cycles of type 2 diabetes. Diabet Med.

[20] Dicker D, Yahalom R, Comaneshter DS, Vinker S (2016). Long-term outcomes of three types of bariatric surgery on obesity and type 2 diabetes control and remission. Obes Surg.

[21] Buchwald H, Estok R, Fahrbach K, Banel D, Jensen MD, Pories WJ, Bantle JP, Sledge I (2009). Weight and type 2 diabetes after bariatric surgery: systematic review and meta-analysis. Am J Med.

[22] Rubio-Almanza M, Camara-Gomez R, Hervas-Marin D, Ponce-Marco JL, Merino-Torres JF (2018). Cardiovascular risk reduction over time in patients with diabetes or pre-diabetes undergoing bariatric surgery: data from a single-center retrospective observational study. BMC Endocr Disord.

[23] Romero Lluch AR, Martinez-Ortega AJ, Socas-Macias M, Jimenez-Varo I, Pereira-Cunill JL, Serrano-Aguayo P, Morales-Conde S, Garcia-Luna PP (2014). Resolution of type 2 diabetes and prediabetes following laparoscopic sleeve gastrectomy: medium term results. Nutr Hosp.

[24] Martin-Rodriguez JF, Cervera-Barajas A, Madrazo-Atutxa A, Garcia-Luna PP, Pereira JL, Castro-Luque J, Leon-Justel A, Morales-Conde S, Castillo JR, Leal-Cerro A (2014). Effect of bariatric surgery on microvascular dysfunction associated to metabolic syndrome: a 12-month prospective study. Int J Obes (Lond).

[25] Ahmed HM, Miller M, Nasir K, McEvoy JW, Herrington D, Blumenthal RS, Blaha MJ (2016). Primary low level of high-density lipoprotein cholesterol and risks of coronary heart disease, cardiovascular disease, and death: results from the multi-ethnic study of atherosclerosis. Am J Epidemiol.

[26] Osto E, Doytcheva P, Corteville C, Bueter M, Dorig C, Stivala S, Buhmann H, Colin S, Rohrer L, Hasballa R (2015). Rapid and body weight-independent improvement of endothelial and high-density lipoprotein function after Roux-en-Y gastric bypass: role of glucagon-like peptide-1. Circulation.

[27] Boido A, Ceriani V, Cetta F, Lombardi F, Pontiroli AE (2015). Bariatric surgery and prevention of cardiovascular events and mortality in morbid obesity: mechanisms of action and choice of surgery. Nutr Metab Cardiovasc Dis.

[28] Raffaelli M, Guidone C, Callari C, Iaconelli A, Bellantone R, Mingrone G (2014). Effect of gastric bypass versus diet on cardiovascular risk factors. Ann Surg.

[29] Patti ME, Houten SM, Bianco AC, Bernier R, Larsen PR, Holst JJ, Badman MK, Maratos-Flier E, Mun EC, Pihlajamaki J (2009). Serum bile acids are higher in humans with prior gastric bypass: potential contribution to improved glucose and lipid metabolism. Obesity (Silver Spring).

[30] Pournaras DJ, Glicksman C, Vincent RP, Kuganolipava S, Alaghband-Zadeh J, Mahon D, Bekker JH, Ghatei MA, Bloom SR, Walters JR (2012). The role of bile after Roux-en-Y gastric bypass in promoting weight loss and improving glycaemic control. Endocrinology.

[31] Risstad H, Kristinsson JA, Fagerland MW, le Roux CW, Birkeland KI, Gulseth HL, Thorsby PM, Vincent RP, Engstrom M, Olbers T (2017). Bile acid profiles over 5 years after gastric bypass and duodenal switch: results from a randomized clinical trial. Surg Obes Relat Dis.

[32] Steinert RE, Peterli R, Keller S, Meyer-Gerspach AC, Drewe J, Peters T, Beglinger C (2013). Bile acids and gut peptide secretion after bariatric surgery: a 1-year prospective randomized pilot trial. Obesity (Silver Spring).

